# Assessment of museum staff exposure to arsenic while handling contaminated exhibits by urinalysis of arsenic species

**DOI:** 10.1186/s12995-017-0173-6

**Published:** 2017-08-25

**Authors:** Amanda Mithander, Thomas Göen, Gitte Felding, Peter Jacobsen

**Affiliations:** 1 Department of Occupational and Environmental Medicine, Copenhagen University, Bispebjerg Hospital, DK-2400 Copenhagen, NV Denmark; 2 Institute and Outpatient Clinic of Occupational, Social and Environmental Medicine, Friedrich-Alexander Universität Erlangen-Nürnberg, Schillerstrasse 25, D-91054 Erlangen, Germany; 3 Chemical Division, Danish Emergency Management Agency, DK-3460 Birkerød, Denmark

**Keywords:** Arsenic, Chemical exposure, Museum objects, Biomonitoring, Species analysis

## Abstract

Preservation of museum objects with inorganic arsenic compounds and contamination of the surroundings has previously been documented. The present study addresses the exposure of museum staff by measuring arsenicals in urine.

After 1 week without exposure, urinary samples were taken before and after handling of preserved skins and analysed by HPLC-ICP-MS for inorganic arsenic, arsenic metabolites and arsenobetaine. The sum of inorganic arsenic and metabolites was an index of exposure. Information about work and seafood intake was obtained by questionnaire.

One out of five subjects had a work-related rise in the exposure index of 18.1 μg As/L to a post-exposure level of 37.1 μg As/L. Four subjects had no certain exposure-related increase in the index.

The study indicates that museum staff may be exposed to arsenic from handling arsenic-preserved objects and supports the use of specified arsenic analysis to avoid interference from organic arsenic.

## Introduction

Employees may be occupationally exposed to hazardous substances by the contact with materials contaminated in previous, eventually not documented processes. Organic objects in ethnological and natural history museums have been treated with toxic preservatives for centuries. Besides other substances, inorganic arsenic compounds have been applied for this purpose and several studies have revealed an arsenic pollution at these workplaces [1–5].

Arsenicals, particularly inorganic arsenic compounds, have chronic health effects including cancer, skin lesions, neurotoxicity and cardiovascular disorders [6, 7]. The most important routes of exposure to arsenic are inhalation and oral uptake. Additionally, some arsenic compounds including inorganic arsenite (As(III)) compounds have shown a limited dermal penetration ability [8, 9]. Therefore measurements of the arsenic air concentration alone may not sufficiently cover the exposure of the employees. In this case, human biomonitoring could be a better way of obtaining a comprehensive and accurate exposure assessment.

There are three different approaches, all of them based on urinary analyses, for human biomonitoring of exposure to arsenic compounds: (a) determination of total arsenic (inorganic plus organic arsenicals); (b) determination of inorganic arsenicals and the main metabolites monomethylarsonic acid (MMA) and dimethylarsinic acid (DMA) by transference to arsine; (c) separation and quantification of different arsenic species, which almost all consist of arsenite (As(III)), arsenate (As(V)), MMA, DMA and arsenobetaine (AsB) [10]. As(III) and As(V) are specific parameters for the uptake of inorganic arsenic, whereas AsB is the most prominent organic arsenic species mostly derived from marine sources. Inorganic arsenicals are, to a great extent, transformed during human metabolism into the methylated species MMA and DMA [11].

The compound-specific analysis (c) thus differentiates between occupational exposure to inorganic arsenicals and dietary exposure to organic arsenicals [12, 13]. The Commission for the Investigation of Health Hazards of Chemical Compounds in the Work Area of the Deutsche Forschungsgemeinschaft has recently published biological reference values based on 95th percentiles of the parameter in a non-occupationally exposed population of working age (Biologischer Arbeitsstoff-Referenzwert, BAR) for arsenite, arsenate, MMA and DMA in urine, thus supporting the evaluation of arsenic species for the assessment of occupational arsenic exposure [14, 15].

Following measurements of very high arsenic concentrations in a historically preserved bird skin of a kind regularly handled by the staff at the Zoological Museum of the University of Copenhagen, the Department of Occupational and Environmental Medicine, Bispebjerg Hospital was engaged in the risk evaluation. The exposure to arsenic was examined using urinary excretion of inorganic arsenic compounds, their methylated metabolites and AsB, before and after handling animal skins presumed to be preserved by arsenicals. To the best of our knowledge, this is the first study which applies a biomonitoring approach to assess the exposure of the employees to this type of exposure.

## Methods and materials

Five employees at the Zoological Museum, involved in handling preserved animal skins were, due to the characteristics of the skins, taken for being potentially exposed to arsenic by the Museums Safety Organization and were included in the study.

After at least 1 week without working with preserved skins, urine specimens were taken before and after a working day’s regular preparation of the exhibit materials without any external intervention. The pre-exposure specimens served as the unexposed control. Both samplings were collected outside the museums premises, after changing clothes and washing hands in order to avoid an external contamination. Information about timing, duration of exposure, personal protection and recent seafood intake were retrieved by a questionnaire.

The urine was analysed for arsenic species at the Institute and Outpatient Clinic of Occupational, Social and Environmental Medicine, University of Erlangen-Nuremberg, Germany by HPLC-ICP-MS according to a procedure proved and published by the Deutsche Forschungsgemeinschaft [16]. Briefly, 100 μL of the urine sample was diluted with the same amount of an aqueous solution containing 1.5% 1 M NH_4_H_2_PO_4_, 0.24% 1 M NaNO_3_, 0.8% 1 M Na-Acetate and 1% ethanol. 50 μL of the mixture was injected into an Agilent 1200 LC system. Separation was performed on a Hamilton PRP-X100 column (250 mm × 4.1 mm, 10 μm) using a gradient from an aqueous 0.5 mM to a 15 mM NH_4_H_2_PO_4_ solution with a flow rate of 1.2 mL/min. Arsenic was quantified in an ICP-MS system (Agilent 7500) with a MicroMist nebulizer and a collision/reaction cell by detecting the mass element 75. The quantitative estimation was performed by external calibration in the range of 0.1–25 μg/L. Samples with results exceeding the upper calibration range were reanalysed after appropriate dilution. For all species (As(III), As(V), MMA, DMA and AsB) the limit of detection (LOD) was 0.07 μg/L and the limit of quantification (LOQ) was 0.2 μg/L. The arsenic concentration was reported as μg arsenic/L urine. The sum of inorganic arsenic and metabolites (As(III), As(V), MMA and DMA) was used as an index of exposure. The creatinine concentration in the spot urine samples was determined by the Jaffé procedure [17]. Only samples, whose creatinine content ranged between 0.3 and 3.0 g/L, were included in the study. The quality of all analyses was proved/ verified by internal quality control measurements as well as by the successful participation in the proficiency tests of the German External Quality Assessment Scheme [18].

The presence of arsenic in the skins was qualitatively analysed in specimens taken from two skins handled by each of the employees on the day of investigation. These analyses were performed by X-ray fluorescence (XRF) and inductively coupled plasma mass spectrometry (ICP-MS) at the Chemical Division, Danish Emergency Management Agency.

## Results and discussion

The five employees handled skins for 2½-3 h without using protective equipment. The presence of arsenic was confirmed by the results of the XRF analyses in all analysed skins, but cannot be quantified.

Post-exposure urinary specimens were taken almost all during the next 2 h after cessation of exposure with the exception of subject 4, who did sampling 5 h after exposure. One of the employees (Subject 1 in Table 1) had eaten seafood the evening before sampling. Another employee (Subject 4) had a daily intake of seafood, including two meals with seafood items between the first and second sample, while none of the remaining three subjects had eaten seafood within 4 days of the sampling.

**Table 1:** Urinary excretion of arsenic species in museum staff before and after handling arsenic-preserved objects. Values in populations without occupational exposure to arsenic compounds and the DFG biological reference value (BAR) are given at the bottom of the table (concentration in μg/L urine)

		As(III)	As(V)	MMA	DMA	AsB	∑iAs+MetAs^b^
Subject 1^a^	pre exp	<LOQ	<LOQ	<LOQ	4.1	92.7	4.1
	post exp	<LOQ	<LOQ	<LOQ	14.7	29.5	14.7
Subject 2	pre exp	<LOQ	<LOQ	0.3	3.1	1.0	3.4
	post exp	<LOQ	<LOQ	<LOQ	1.6	0.8	1.6
Subject 3	pre exp	<LOQ	<LOQ	<LOQ	5.9	17.1	5.9
	post exp	<LOQ	0.3	0.5	5.5	14.0	6.3
Subject 4^a^	pre exp	<LOQ	<LOQ	<LOQ	23.7	36.4	23.7
	post exp	<LOQ	<LOQ	<LOQ	21.5	24.7	21.5
Subject 5	pre exp	1.8	0.2	3.3	13.7	8.1	19.0
	post exp	2.3	<LOD	9.8	25.0	8.0	37.1
Population studies							
Germany [18]	geo mean; 95perc.	0.2; 0.7	0.1; 1.1	0.4; 2.3	3.0; 15.8	1.6; 23.3	-
UK [19]	median; 95perc.	0.1; 0.7	<0.03; 0.2	0.6; 2.0	2.9; 10.7	6.6; 115.7	- -
Reference value (BAR) [14]		0.5	0.5	2	10	-	14

^a^Subjects with seafood intake during survey or within 24 h before sampling

^b^Sum of As(III), As(V), MMA and DMA

The results of the urinary analyses are displayed in Table 1, which also includes data from two population-based studies and the German reference value for comparison [19, 20].

The concentration of inorganic arsenic (As(III) and As(V)) was below LOQ in 16 out of 20 analyses and showed no clear increase in the post-exposure concentration in any of the five subjects.

The metabolite MMA was detected in four out of ten samples including both samples of Subject 5 with a post-exposure increase of 6.5 μg/L. Additionally, MMA was found in low concentration in the pre-exposure sample of Subject 2 and the post-exposure sample of Subject 3.

DMA, the major metabolite of inorganic arsenic, was detected in both pre- and post-exposure samples of all five individuals. For Subjects 1 and 5 the DMA concentration increased from pre- to post-exposure samples by 10.6 and 11.3 μg/L urine respectively. The remaining three subjects had DMA concentrations at a lower level and without post-exposure increase.

Arsenobetaine was found in high concentrations in urine from subject 1 and 4, in accordance with seafood intake within 24 h of the two samplings. For three subjects without recent seafood intake, urinary AsB concentration was at a low or moderate level.

**Fig. 1:**
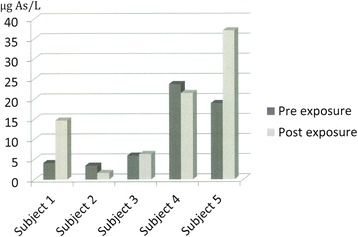
Urinary concentrations of the sum of As(III), As(V), MMA and DMA in urine of five subjects before and after handling of arsenic preserved skins

The sum of inorganic arsenic and metabolites (index of exposure) showed an increase in Subject 1 and 5 at 10.6 and 18.1 μg/L, reaching a post-exposure level of 37.1 μg/L for Subject 5, Fig. 1. This subject also showed an increase in the single compounds included in the index, except for As(V), Table 1.

The result strongly suggests a certain exposure to arsenic for subject 5 by handling preserved skins. After about 3 h of work, the post-exposure value exceeded the US Biological Exposure Index (BEI) for occupational exposure to inorganic arsenic at 35 μg arsenic/L but not the German guidance value at 50 μg arsenic/L [14, 21].

The exposure-related increase in the exposure index of subject 1 at 10.6 μg/L was solely due to an increase in the metabolite DMA. This person had eaten seafood the evening before sampling, which was reflected in a high level of arsenobetaine especially in the first sample. It cannot be excluded that the rise in DMA was caused by seafood intake since some organic arsenicals in seafood may be metabolised to DMA [22–24]. For the three remaining subjects no exposure-related increase in the urinary concentrations of arsenicals was found.

Inorganic arsenic and MMA levels were low for all subjects, except for Subject 5, and for most analyses below the LOQ of 0.2 μg/L. These results were at a level comparable to values in European general population studies indicating no or a not detectable additional exposure to inorganic arsenic in these subjects, see Table 1 [19, 20].

DMA is the major metabolite of inorganic arsenic, but has, as mentioned before, also been associated with metabolism of organic arsenic compounds in seafood. This component exceeded the German reference value (BAR) in both samples of Subject 5. The two subjects with recent seafood intake also presented values above the BAR concomitant with inorganic arsenic and a MMA urinary concentration below LOQ. These results are both in concert with the established metabolism of inorganic arsenic into DMA and with experimental findings that certain organic arsenic in seafood may provide or be metabolized to DMA [22–25].

The results support a moderate uptake of arsenic in one subject who dealt with museum objects definitely containing arsenicals and who wasn’t using protective equipment. No indication of exposure to arsenic from the preserved skins was found for four subjects. An explanation might be that the actually handled skins or some of them contained less arsenic than had been previously found; an explanation which cannot be verified since the arsenic content of the skins was not quantified. The handling of the skins may also have been safer than assumed, but this explanation isn’t in concert with the rapid increase in the exposure index of Subject 5 exceeding the US Biological Exposure Index after few hours of handling skins. Therefore, we recommend a systematic review of the prevention measures considering the reasonable use of respiratory and dermal protection as well as the consequent separation of operation and break areas.

The main limitations of the study are the limited number of individuals involved and the lack of quantitative measurements of arsenic in the handled skins. These two limitations are due to the study being conducted “per occasionem”, i.e. as part of the routine prevention surveillance.

Organic objects in museum collections are a potential source of arsenic exposure, which has received limited attention. The present study indicates that the risk of high exposure to inorganic arsenic from preserved skin is limited. However, the study has limited potential to evaluate the exposure in detail. The clear increase in urinary inorganic arsenic and metabolites detected in the post-exposure urine samples of one of the investigated subjects makes it an issue that ought to be evaluated in depth in further studies. In any case, the study supports specified analyses of arsenicals in order to avoid incorrect exposure measurements from interfering organic arsenicals in seafood.
